# Conventional Culture Conditions Limit the Ability of Bovine Mammary Epithelial Cells to Produce Milk Fat

**DOI:** 10.3390/foods15183234

**Published:** 2026-09-13

**Authors:** Noam Tzirkel-Hancock, Christina Ramires Ferreira, Roni Tadmor-Levi, Nurit Argov-Argaman

**Affiliations:** 1 The Robert H. Smith Faculty of Agriculture, Food and Environment, The Hebrew University of Jerusalem, P.O. Box 12, Rehovot 761001, Israel; noam.tzirkel@mail.huji.ac.il (N.T.-H.); roni.tadmor@mail.huji.ac.il (R.T.-L.); 2 Bindley Bioscience Center, Purdue University, West Lafayette, IN 47907, USA; cferrei@purdue.edu

**Keywords:** mammary epithelial cells, fatty acid synthesis, bovine milk fat, cellular agriculture, lipid metabolism, MRM profiling

## Abstract

Bovine milk fat is distinguished by a high proportion of short and medium chain fatty acids, a profile that mammary epithelial cells (MEC) fail to reproduce in vitro. When considering culturing MEC as a source for alternative dairy products, this failure limits the ability of replicating the functionality of milk fat. Conventional media used to grow the cells is designed to mimic monogastric animal plasma, disregarding the profound metabolic differences compared with ruminant plasma. Therefore, we hypothesized that the inability to reproduce milk fat composition in vitro reflects substrate composition rather than an intrinsic limitation of the cells. To test this, we gradually modulated MEC media composition towards a ruminant-like substrate balance, lowering glucose and supplementing acetate. Pathway analysis revealed that high glucose media enhanced TCA cycle without activating de novo lipogenesis, while acetate increased lipid accumulation in both glucose backgrounds. Caprylic acid (C8:0), a medium-chain fatty acid characteristic of bovine milk fat, increased with acetate supplementation and peaked under low glucose media with acetate, consistent with acetate increasing lower molecular weight triglycerides. These results indicate that the limited capacity of MEC cultures to generate milk-like fatty acid profiles is substantially influenced by the substrate environment, and that a ruminant-like shift in substrate availability partially, but not completely, redirects fatty acid output towards the milk fat profile.

## 1. Introduction

MEC in vitro models are widely used to study the molecular and metabolic processes of bovine milk fat synthesis [1,2] and to explore the possibility for “cultured milk” production as an alternative production platform for bovine milk sustainable alternatives [3,4]. However, although these models provide substantial insight into the biochemical and molecular machinery of lactation, they fail to reproduce the fatty acid composition characteristic of bovine milk fat [5].

Fat synthesis is a highly conserved process in mammalian physiology, requiring the coordinated expression of multiple enzymes involved in uptake and activation of lipogenic building blocks, fatty acid synthesis, and triglyceride assembly [6]. Although conserved throughout evolution, the milk of different mammalian species consists of specific fatty acid compositions that reflect both evolutionary adaptations and metabolic constraints. Ruminant milk, which is the major global source of dairy products, possesses a unique fatty acid profile compared to other mammalian milk, containing approximately 25% short and medium-chain fatty acids (C4:0–C14:0) synthesized de novo within the mammary epithelial cells (MEC). Bovine milk fat composition differs from other lipogenic tissues such as bovine adipose which consists of only 2.5% medium-chain fatty acids and no short-chain fatty acids [7] and is fundamentally different from vegetable oils which usually have high portions of long unsaturated fatty acids [8]. Efforts have been made over the last decade to replace milk fat with non-animal fat sources due to welfare and environmental considerations. However, the limited ability to replicate bovine milk fat in other organisms, tissues, or in ex vivo settings, has become a major hurdle in the production of high quality, tasty and safe dairy replacers [9]. All currently available milk fat replacers possess considerably different fatty acid compositions compared to dairy fat, which leads to inferior technological and rheological properties, with limited ability to mimic the texture, mouthfeel and overall consumption experience of real dairy products. Therefore, a deep understanding of the mechanism controlling milk fat synthesis in ruminants is required in order to implement the data in other lipid producing systems and to control the fatty acid and lipid profile produced.

In all organisms, the central enzyme responsible for de novo fatty acid synthesis is fatty acid synthase (FASN), a multienzyme with seven catalytic sites, which are all encoded within one gene in mammals [10]. Since only one FASN transcript is active in all bovine lipogenic tissues [11], it seems the abundance of short and medium chain fatty acids in ruminant milk is not attributed to the distinct properties of the ruminant FASN. Rather, it seems that broader, unique production capabilities specific to the mammary gland enable this fatty acid profile.

To explore the possibility that changes in the pathway rather than in a single enzyme are responsible for the unique fatty acid profile of bovine milk, a mega-analysis was conducted and compared expression patterns of lipogenic genes between the bovine mammary gland, milk cells and adipose tissue [12]. Variations were found in genes contributing lipogenic building blocks to FASN and in genes encoding to enzymes which activate and utilize the products of FASN in order to incorporate them into more complex lipids like triglycerides and polar lipids. More specifically, acyl-CoA synthase short-chain family member 1 (ACSS1), which activates acetate to acetyl-CoA which can then be utilized by FASN for fatty acid synthesis was uniquely enriched in the mammary gland and not in other lipogenic tissues.

In ruminants, unlike in monogastric animals, the source of acetyl-CoA as a substrate for FASN is acetate [13] and its concentration in the plasma is altered throughout lactation [14], along with changes in fatty acid composition in milk [15]. Using an in vitro model, bovine MEC were shown to be highly sensitive to acetate compared with other cell types, responding to acetate with increased medium-chain fatty acid concentrations and overall triglyceride accumulation. This was supported by coordinated upregulation of ACSS1 and diacylglycerol acyltransferase (DGAT) gene expression. This acetate-responsive phenotype was not observed in fibroblasts, highlighting the mammary-specific nature of these metabolic adaptations [16].

The high responsiveness of MEC to acetate is aligned with the fact that acetate concentrations in ruminants’ plasma is high compared with monogastric plasma [14]. The concentration of glucose in ruminant plasma is lower and volatile fatty acids like acetate are higher compared with monogastric animals [17,18]. However, standard medium used to culture MEC does not reflect this unique composition andis designed to simulate plasma of monogastric animals Another significant metabolic difference which may contribute to the differences between in vivo and in vitro MEC output is lactose synthesis and the source of acetyl CoA used for fatty acid synthesis. In the mammary gland in vivo glucose is primarily used for lactose synthesis. However, in vitro, MEC lines in culture exhibit limited lactose synthesis capacity due to low α-lactalbumin expression [19]. This implies that glucose is not channeled toward lactose synthesis as occurs in vivo [20] and is instead used for alternative pathways such as glycolysis. In vivo, the acetyl-CoA produced from pyruvate, the glycolytic product of glucose, serves only as a minor source of carbons directed towards de novo fatty acid synthesis, due to low expression and activity of ATP-citrate lyase (ACLY) in the ruminant mammary gland [21]. Instead, ruminants primarily rely on plasma acetate, derived from rumen fermentation of structural carbohydrates, for de novo fatty acid synthesis [22]. Thus, the standard culture media often applied to ruminant MEC likely change the metabolic capacity of cells to synthesize short and medium chain fatty acids.

The present study assessed how glucose and acetate availability shape MEC metabolism and lipid output. The study design was intended to shift the balance of the two principal lipogenic carbon sources in the direction that distinguishes ruminant from monogastric plasma, and to understand whether such a shift is sufficient to alter MEC lipid output towards a milk-like profile. Semi-targeted lipidomics and metabolomics analysis together with monitoring the lipogenic capacity of the cells allowed us to dissect the contributions of substrate availability to MEC metabolism and specifically the effect on lipid metabolism.

## 2. Materials and Methods

### 2.1. Cell Culture and Study Design

Mac-T were purchased from Bet HaEmek, Biological Industries, Bet HaEmek, Israel. For experiments, cells were seeded in 10 mL plastic dishes with growth medium consisting of DMEM F-12, 10% FBS, 1% L-Glutamine and antibiotics all purchased from Biological Industries (Beit Haemek, Israel) and 0.01% cortisol and 0.01% insulin (Sigma Aldrich Ltd., Rehovot, Israel). Upon reaching confluence, cells were treated with Trypsin–EDTA solution C (Biological Industries, Beit HaEmek, Israel) and re-plated depending on the experiment performed. For experiments, confluent cells were exposed to serum-free growth medium for 24 h in a 2 × 2 factorial arrangement of two factors: glucose concentration (high glucose DMEM, HG, 3000 mg/L, or low glucose DMEM, LG, 1000 mg/L) and acetate supplementation (0 or 40 mM sodium acetate, diluted directly into the growth medium). This gave four treatments: HG, HG + Ac, LG, and LG + Ac. All other medium components were identical across treatments. All reagents were purchased from Sigma Aldrich LTD, Rehovot, Israel.

Dose and time response experiments were performed in order to determine the concentration of acetate to use in the treatments (Supplementary Figure S1). Hormonal treatments did not include prolactin, in order to reduce the inconsistent role played by the hormone in bovine lactation and lipid synthesis in the mammary gland. Previous experiments conducted with or without 48 h exposure to prolactin to allow differentiation prior treatment commencement showed a similar response in terms of triglyceride accumulation and relevant gene expression (Supplementary Figure S2).

### 2.2. Nile Red Staining and Flow Cytometry

Cells were seeded at 15,000 cells per ml in 30-mm petri dishes. Treatment media was supplemented with 360 µM of oleic acid. Post-treatment cells were detached using 0.25% trypsin-EDTA C (Sigma Aldrich Israel Ltd. Rehovot, Israel) and pelleted by centrifugation. Cell pellets were washed twice in PBS, then fixed with 4% paraformaldehyde for 20 min at room temperature and washed twice more with PBS. Cellular lipid droplets were stained with Nile Red (1 µg/mL) for 15 min at room temperature under dark conditions. Following two additional PBS washes, cells were resuspended in PBS for flow cytometric quantification.

Cell suspensions were transferred to 96-well plates and analysed on an Accuri C6 flow cytometer (BD Biosciences, Franklin Lakes, NJ, USA) using 488 nm excitation. Nile Red fluorescence intensity was detected in the FL2 channel (585/40 nm) as an indicator of cellular lipid content. A minimum of 10,000 events were acquired per sample, with total event counts used for cell enumeration. Flow cytometry data were processed using BD Accuri C6 Plus Software.

### 2.3. Lipidomics and Metabolomics Multiple Reaction Monitoring (MRM) Profiling

Dried lipid extracts were resuspended in 200 µL of acetonitrile:methanol:300 mM ammonium acetate 3:6.65:0.35 (*v*/*v*/*v*) to generate stock solutions. Next, the stock was diluted with the same solvent mixture and tested for optimal ion signal. For data acquisition, the working solvent will be spiked with 0.05 ng/µL of each lipid contained in the internal standard mixture (Equisplash, Avanti Polar Lipids, Alabaster, AL, USA). Metabolites were diluted in 200 µL of acetonitrile:water 1:1 (*v*/*v*) and further dilution was performed in acetonitrile:methanol:300 mM ammonium acetate 3:6.65:0.35 (*v*/*v*/*v*) for sample injection. The volume of 12 µL of each sample was used for each injection. Lipidomic and metabolomic analyses were performed by MRM profiling, a direct-injection mass spectrometry strategy that surveys small molecules and lipids at the class/species level without chromatographic separation, following the workflow established previously [23]. Briefly, the approach comprises two phases. In the discovery phase, pooled samples are interrogated for class-diagnostic ion transitions (MRMs), in which the precursor ion corresponds to the species-level m/z of the lipid or metabolite. The lipid ion transition lists were built from the LIPID MAPS Structure Database and polar metabolites ion transitions were surveyed from the literature [24,25]. Since MRM profiling is a semi-targeted screening approach rather than a structurally confirmatory one, we previously validated a defined subset of discriminating features using an orthogonal rigorous approach, narrowing the candidate list by retention time [26]. Moreover, multiple lipid compounds were validated by LC-MS/MS with an authentic internal standard [27]. Structural confirmation was validated by authentic standard co-injection for a targeted subset of metabolites and lipids. Data is reported as relative ion intensity as previously describe [28].

For the lipids, in the subsequent screening phase, individual samples were interrogated only for the transitions detected above background in the discovery phase, with a transition considered detected when its maximum intensity exceeded a control to blank ratio threshold of 1.5, relative to a blank extraction. Data was acquired on an Agilent 6410 triple-quadrupole mass spectrometer (Santa Clara, CA, USA) equipped with a G1367A 1100-series autosampler operating at a flow rate of 8 µL/min and a pressure of 100 bar. The samples were electro-sprayed directly into the ion source with a capillary voltage of 4.0 kV in positive ion mode and 3.5 kV in negative ion mode, a dwell time of 25 ms, a gas flow of 5.1 L/min at 300 °C, and an electrospray source temperature of 300 °C. Between sample injections, acetonitrile containing 0.1% formic acid was used as the wash solvent.

Triacylglycerols (TG) and diacylglycerols (DG) were annotated according to their total carbon number, total number of double bonds, and the identity of one fatty chain (i.e., TG 34:2_16:0). TG species were then grouped into molecular weight categories by total carbon number, into low (less than 34 carbons), medium (35 to 42 carbons), and high (more than 44 carbons) categories.

### 2.4. Statistical Analysis

Statistical analysis of both the lipidomic and metabolomic datasets was performed in MetaboAnalyst 6.0. Prior to analysis, the data of each metabolite/lipid species was normalised to its blank reading. For the metabolomics data, principal component analysis (PCA) was used to visualize overall separation among the four treatment groups. Pairwise differences were assessed and metabolites exceeding a fold-change threshold of 1.5 and a *p*-value lower than 0.05 were considered significantly altered and were submitted to pathway analysis. Enrichment and topology analysis was carried out using the KEGG pathway library for *Bos taurus*. A discovery threshold of FDR ≤ 0.05 was applied for the enrichment analysis. Supervised classification was performed by random forest (1000 trees), both across all four treatment groups simultaneously and for each pairwise comparison. For the lipidomics data, group separation and discriminant features were evaluated by partial least squares-discriminant analysis (PLS-DA) and random forest across all four treatment groups. For TG molecular weight analysis, Homogeneity of variance was assessed by Bartlett’s test (*p* > 0.05) followed by a two-way ANOVA performed with glucose, acetate, and their interaction as fixed effects. When a significant glucose × acetate interaction was detected, Student’s t-tests were used to compare treatments within each level of the other factor. Where lipid classes were below the limit of detection, the two-way ANOVA was not applicable to this lipid class, therefore we analysed the simple effects by the non-parametric Wilcoxon rank-sum test. Significance was declared at *p* ≤ 0.05.

## 3. Results

### 3.1. Metabolomics Analysis of MEC Under Varying Acetate and Glucose Concentrations

To assess how substrate availability affects the MEC metabolome, confluent Mac-T cells were treated for 24 h with one of four conditions: high glucose (HG; standard media), high glucose with 40 mM acetate (HG + Ac), low glucose (LG), and low glucose with 40 mM acetate (LG + Ac). Of these four, LG + Ac is the closest in composition to the substrate balance of ruminant plasma. Following treatment, cells were harvested and analysed for small-metabolites and lipids. 

**Figure 1:**
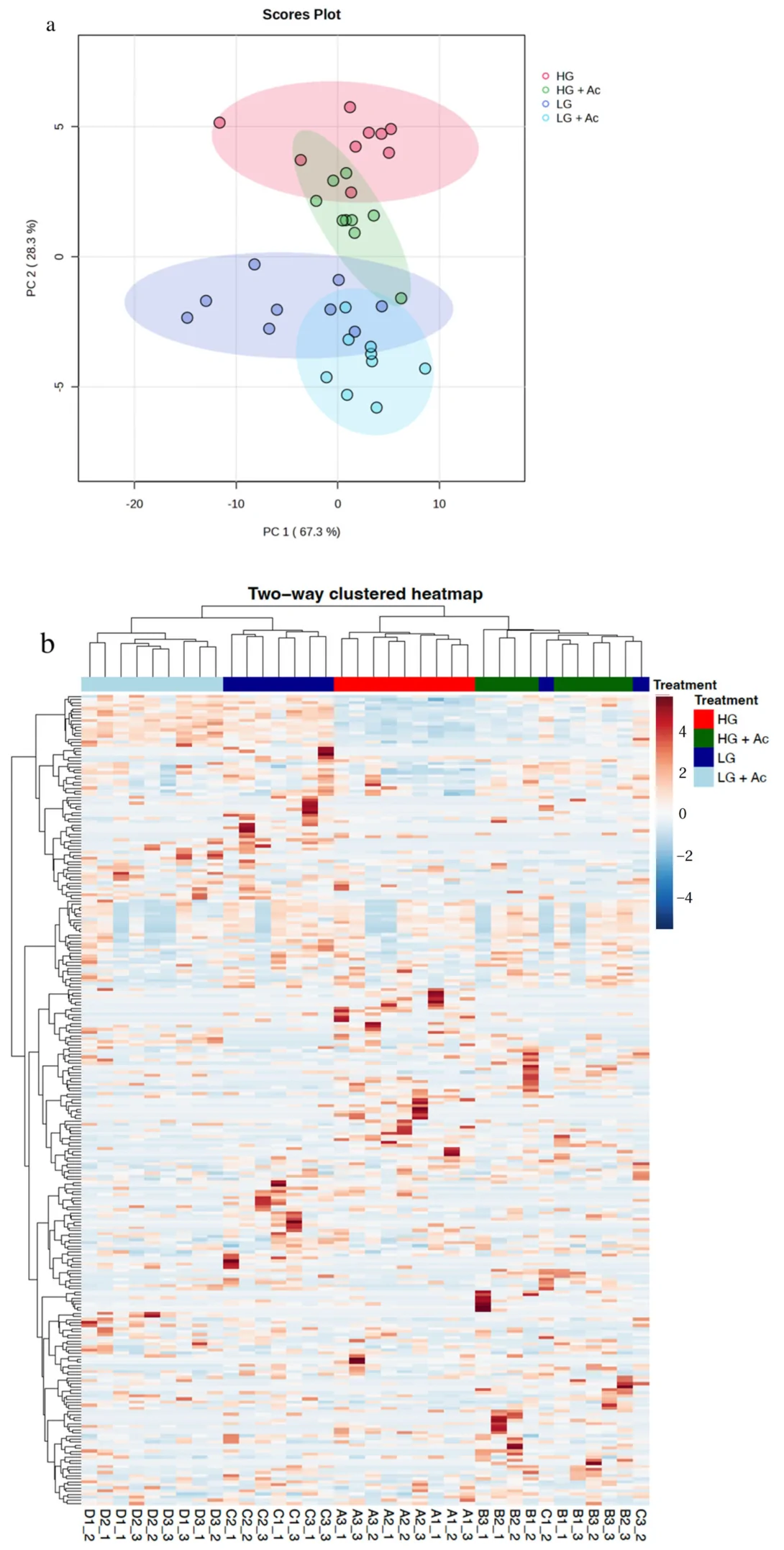
Metabolomics analysis of MEC following 24-h treatment under four conditions: high glucose (HG; red), high glucose with 40 mM of acetate (HG + Ac; green), low glucose (LG; blue) or low glucose with 40 mM of acetate (LG + Ac; light blue). (**a**) PCA based on metabolite concentration of MEC samples under the four treatments. (**b**) Two-way hierarchical clustering and heat map of all metabolites measured in MEC under the four treatments. Colour scale represents the relative concentration value-red indicates a higher concentration and blue indicates a lower concentration.

Principal component analysis (PCA) of metabolite concentrations showed partial treatment related structuring, with component 1 (PC1) accounting for 67.3% of the variance and component 2 (PC2) accounting for 28.3% of the variance (Figure 1a). Samples were primarily separated by glucose level along PC2, with HG and HG + Ac clustering apart from LG and LG + Ac. Within each glucose background, acetate-treated samples were partly separated from their respective controls. The greatest separation was observed between HG and LG + Ac samples.

Hierarchical clustering based on metabolites readout broadly supported the PCA pattern, with samples separating largely by glucose level. Within each glucose background, acetate supplementation appeared to contribute additional sub-clustering, indicating a secondary effect of acetate availability on the MEC metabolome (Figure 1b).

### 3.2. Metabolic Pathway Enrichment Analysis in MEC Under Metabolic Manipulations

**Figure 2:**
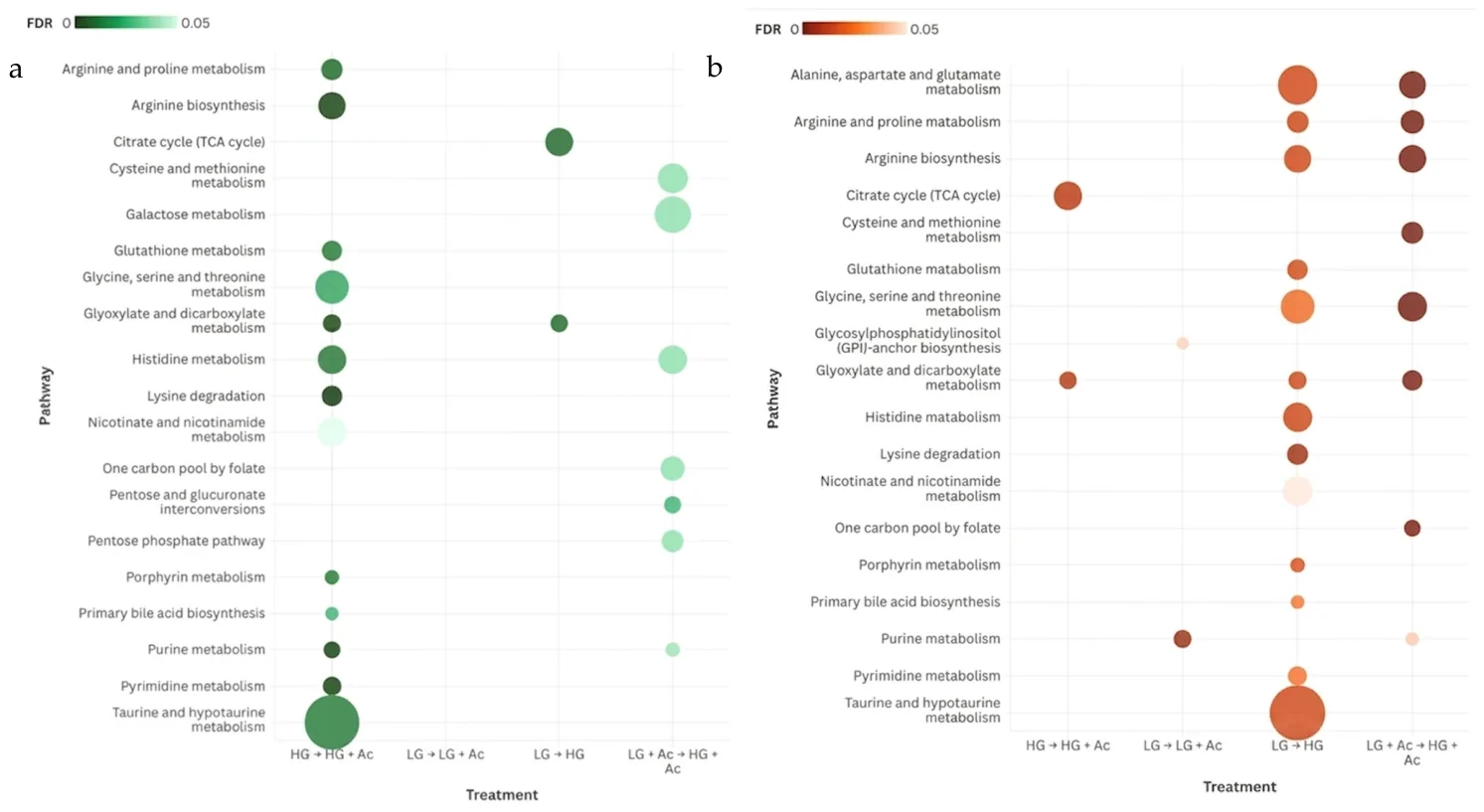
Dot plots showing KEGG pathways significantly enriched among (**a**) upregulated and (**b**) downregulated metabolites in each treatment: HG + Ac (high-glucose medium supplemented with acetate vs. high-glucose control), LG + Ac (low-glucose medium supplemented with acetate vs. low-glucose control), HG vs. LG (high-glucose vs. low-glucose control) and HG + Ac vs. LG + Ac (high glucose with acetate vs. low glucose with acetate control). Pathway analysis was performed in MetaboAnalyst using KEGG Bos taurus library. Circle size represents pathway impact and colour intensity represents the false discovery rate (FDR), with darker shades indicating lower (more significant) FDR values as shown in the colour scales (0–0.05). Only pathways with FDR ≤ 0.05 are displayed.

To identify the metabolic pathways most affected by glucose and acetate concentration in bovine MEC, we performed pathway enrichment analysis using MetaboAnalyst on metabolites significantly altered by each treatment (HG + Ac versus HG, LG + Ac versus LG, HG versus LG and HG + Ac versus LG + Ac). Enriched pathways were separated by direction of change into upregulated (Figure 2a) and downregulated (Figure 2b) sets.

Regarding the upregulated pathways (Figure 2a) acetate supplementation had a marked effect under high glucose (HG → HG + Ac) but none under low glucose. Acetate with a high glucose background upregulated a broad set of pathways associated with amino acids metabolism (including taurine, hypotaurinearginine biosynthesis, glycine/serine/threonine metabolism, histidine metabolism and lysine degradation), while only pathways that were downregulated were the TCA cycle, glyoxylate and dicarboxylate metabolism (Figure 2b). In contrast, acetate in the low glucose background (LG → LG + Ac) produced essentially no upregulated pathways, with the downregulation of only glycosylphosphatidylinositol anchor biosynthesis and purine metabolism.

The effect of raising glucose concentration was also dependent on acetate background. Without acetate (LG → HG), high glucose upregulated the TCA cycle and the glyoxylate and dicarboxylate metabolism pathways. Howevera large number of pathways were downregulated–mostly amino acids metabolism (e.g., taurine and hypotaurine metabolism, alanine/aspartate/glutamate,, glycine/serine/threonine, histidine, arginine and proline metabolism, arginine biosynthesis just to name a few) but alsoTCA cycle, glutathione metabolism, nicotinate and nicotinamide metabolism as well as the one carbon pool by folate pathways. With acetate present in both glucose concentrations (LG + Ac → HG + Ac), high glucose most strongly upregulated galactose metabolism, along with amino acid metabolism (including cysteine, methionineand histidine metabolism), in addition to increase in energy and reducing elements pathways like one-carbon pool by folate and pentose phosphate pathway. Pathways reduced by high glucose under acetate background include amino acid metabolism (including arginine and proline and lysine degradation). Notably, cysteine and methionine metabolism, one-carbon pool by folate and purine metabolism each appeared in both the upregulated and downregulated sets for this comparison, indicating divergent changes among individual metabolites within the same pathway.

### 3.3. Random Forest Analysis Identifies Distinct Metabolite Signatures Associated with Acetate Supplementation and Glucose Availability

**Figure 3:**
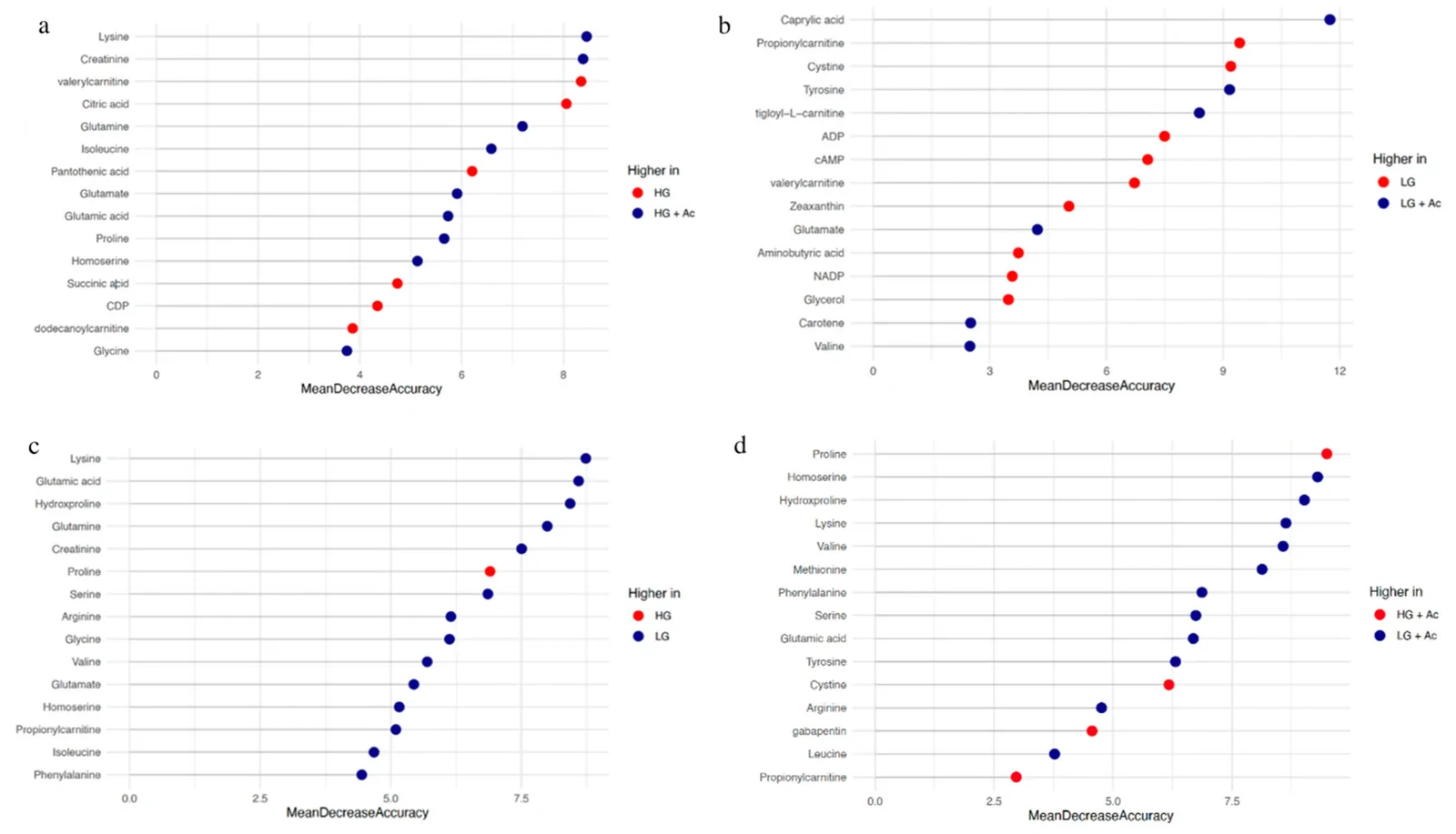
Top 15 most discriminating metabolites identified by Random Forest analysis for four comparisons: (**a**) HG versus HG + Ac. (**b**) LG versus LG + Ac. (**c**) HG versus LG. (**d**) HG + Ac versus LG + Ac. For each comparison, features are ranked by Mean Decrease in Accuracy (*x*-axis), which quantifies the loss in classification accuracy when each feature’s values are randomized. Higher values indicate greater importance in classification. The coloured panel adjacent to each plot shows the relative abundance of each metabolite in the two compared groups, with values z-score normalised per metabolite (red = high, blue = low).

To identify the metabolites most strongly discriminating between treatments, Random Forest classification was applied to four pairwise comparisons (Figure 3). The classifier achieved an out-of-bag (OOB) error rate of 5.6% for HG versus HG + Ac (Figure 3a) and 0% for LG versus LG + Ac (Figure 3b) HG versus LG (Figure 3c) and HG + Ac versus LG + Ac (Figure 3d), indicating robust separation of the metabolome between treatment conditions.

In the HG versus HG + Ac comparison (Figure 3a), lysine and creatinine were the most discriminating features that were higher with acetate supplementation, while valerylcarnitine and citric acid were the most discriminating features that were higher without acetate. Additional discriminating metabolites elevated by acetate included, glutamine, isoleucine, glutamic acid, and proline. Fewer additional discriminating metabolites were higher without acetate in the media, including pantothenic acid, succinic acid, CDP and dodecanoylcarnitine.

Caprylic acid was the strongest discriminator between LG and LG + Ac with higher availability with acetate supplementation (Figure 3b). Besides caprylic acid, there were fewer discriminating metabolites which were higher with acetate, these included tyrosine and tigloyl-L-carnitine and to a lower extent also glutamate, carotene and valine. Notably, several acylcarnitines (propionylcarnitine and valerylcarnitine) and nucleotide-related metabolites (ADP, cAMP, NADP) were higher when acetate was not included in the media.

Comparison of the two glucose levels without acetate (HG → LG; Figure 3c) revealed that nearly all top-ranked metabolites, including lysine, glutamic acid, hydroxyproline, glutamine, creatinine, serine, arginine, glycine, valine, glutamate, homoserine, propionylcarnitine, isoleucine, and phenylalanine, were elevated in low glucose media, with only proline higher in high glucose media.

Interestingly, proline was also the top discriminator in the comparison of glucose levels with acetate in the background (HG + Ac to LG + Ac; Figure 3d), with higher levels in HG + Ac. Additional discriminators that were higher in HG + Ac were cystine, gabapentin and propionylcarnitine. Similarly to the glucose comparison without acetate, many of the discriminators between glucose levels with acetate were amino acids, that were elevated in the low glucose background, including lysine, proline, methionine, phenylalanine, serine, glutamine, tyrosine, arginine and leucine.

### 3.4. Triglyceride Content Differs Between Treatments

**Figure 4:**
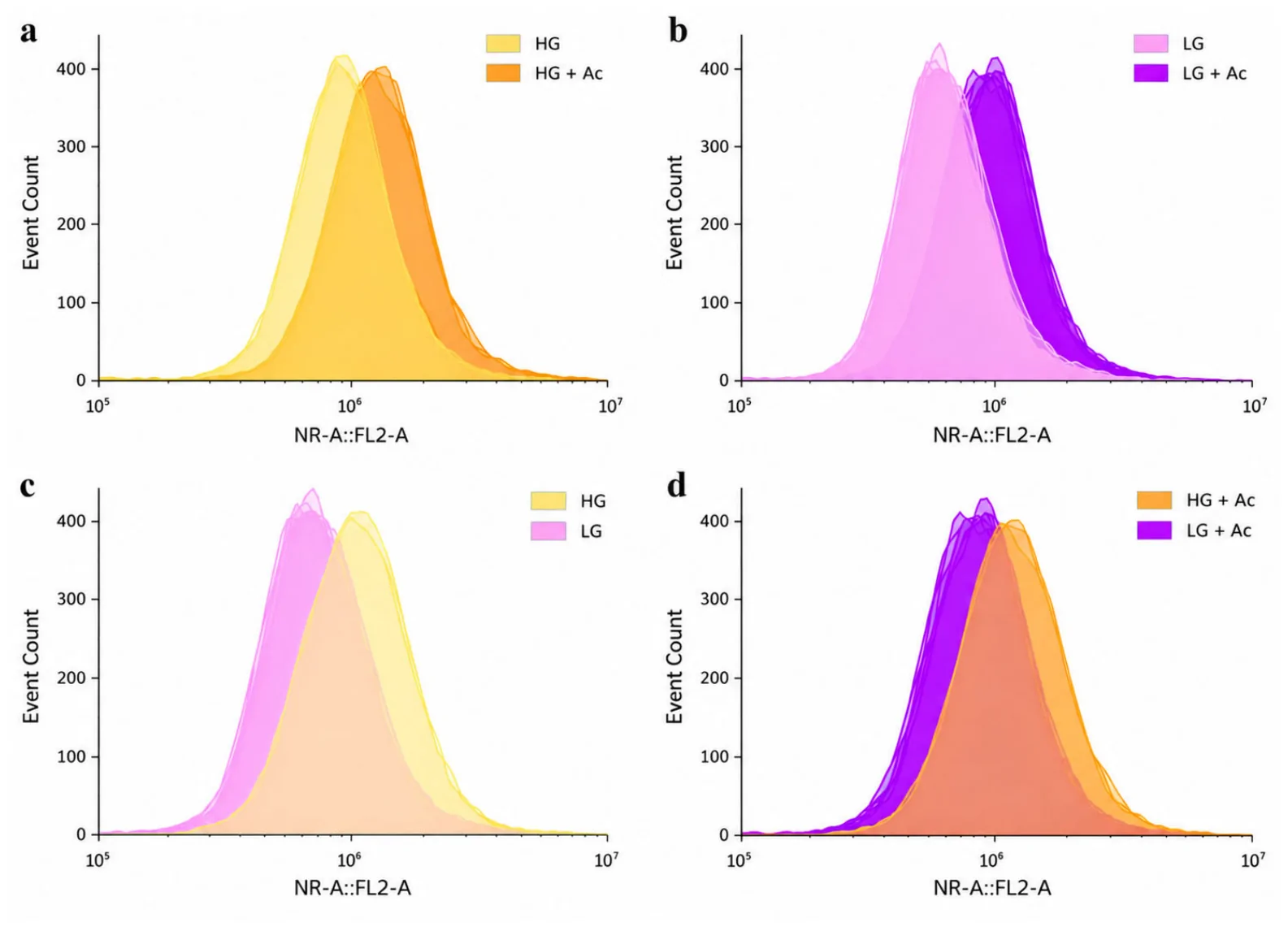
Flow cytometry analysis of Nile Red fluorescence in MEC. *X*-axis: FL2 channel representing Nile Red intensity (lipid content); *Y*-axis: event count. Comparisons were made between (**a**) HG and HG + Ac. (**b**) LG and LG + Ac. (**c**) HG and LG. (**d**) HG + Ac 1and LG + Ac.

Intracellular triglyceride content in MEC was estimated by the intensity of hydrophobic dye (Nile Red) incorporated into lipid droplets. Acetate treatment increased lipid droplet accumulation in MEC both when added to HG (Figure 4a) and LG media (Figure 4b) inducing a clear shift in fluorescence intensity indicating a significant increase in intracellular lipid content. The addition of glucose also induced an increase in fluorescence, regardless of acetate availability (Figure 4c,d).

### 3.5. Lipidomics Analysis

To further understand how glucose and acetate availability affect MEC fatty acid synthesis, we performed lipidomic profiling of MEC cultured under the four treatments (HG, HG + Ac, LG, LG + Ac) and analysed the data using multivariate approaches.

**Figure 5:**
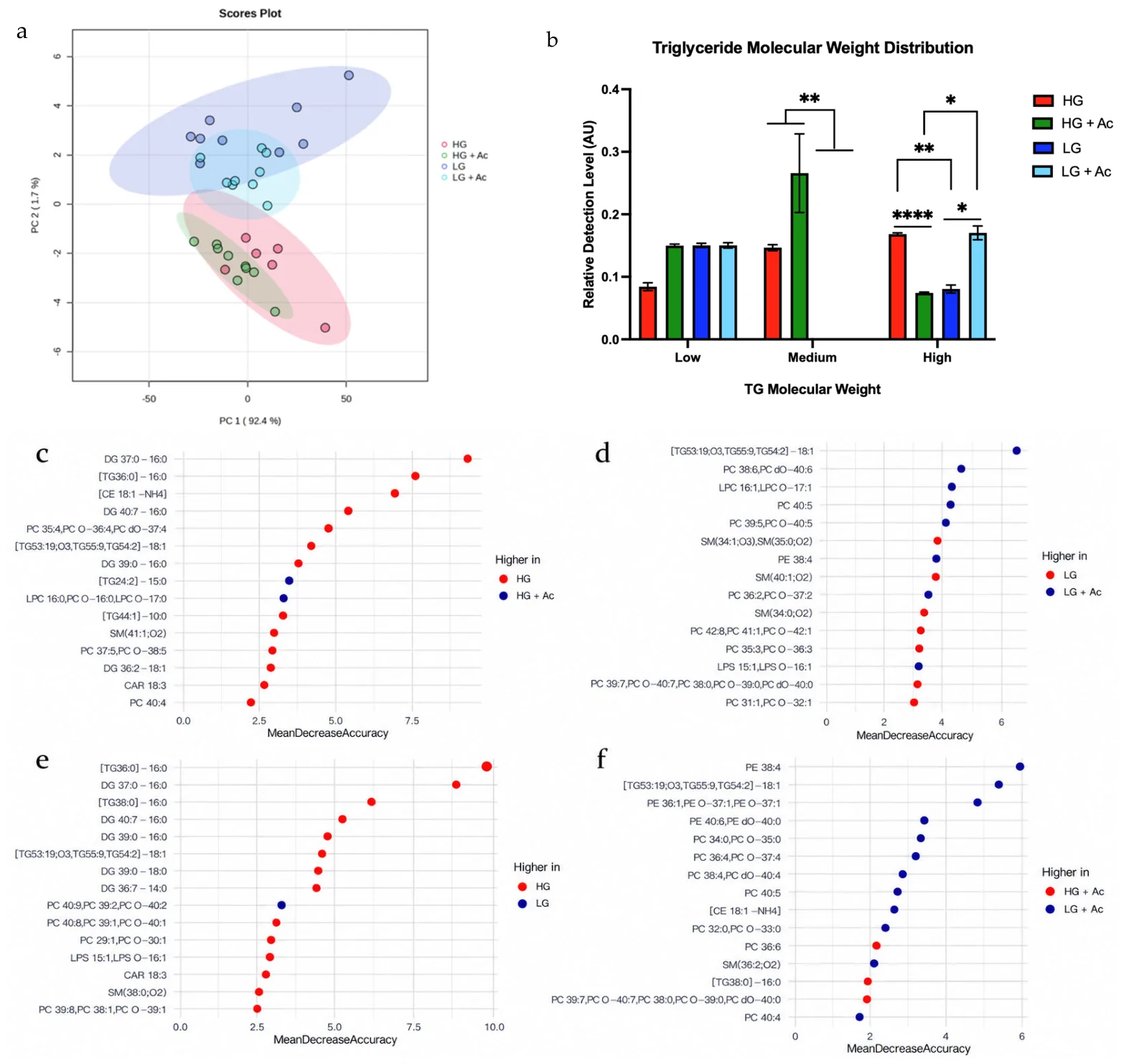
(**a**) PLS-DA of lipid species concentrations in MEC. (**b**) Relative detection level (AU) of triglyceride species based on molecular weight (low, medium, high). Bars represent mean ± SEM. Significant differences are indicated by asterisks (* *p* < 0.05, ** *p* < 0.01, *** *p* < 0.001, **** *p* < 0.0001) (**c**–**f**) Top 15 most discriminating lipid species identified by Random Forest analysis for four comparisons: (**c**) HG versus HG + Ac. (**d**) LG versus LG + Ac. (**e**) HG versus LG. (**f**) HG + Ac versus LG + Ac. For each comparison, features are ranked by Mean Decrease in Accuracy (*x*-axis), which quantifies the loss in classification accuracy when each feature’s values are randomized. Higher values indicate greater importance in classification. The coloured panel adjacent to each plot shows the relative abundance of each metabolite in the two compared groups, with values z-score normalised per metabolite (red = high, blue = low).

A PLS-DA of the full lipidomic dataset was used to evaluate treatment discrimination among MEC samples (Figure 5a). The first component accounted for 92.4% of the model variation and the second component accounted for 1.7%, with treatment-related partial separation most apparent along the second component. Samples were primarily separated according to glucose availability, with HG and HG + Ac samples grouping apart from LG and LG + Ac samples. Within each glucose background, acetate-treated samples showed partial clustering apart from their respective controls.

We separated TG species into three groups based on molecular weight-high, medium and low molecular weight (Figure 5b). Low molecular weight triglycerides were ~1.5 fold higher in all treatment compared to HG, although not statistically significant (*p* = 0.143). Glucose availability was the primary determinant of medium molecular weight triglyceride abundance (*p* = 0.0039)-these species were below the limit of detection in both low glucose treatments, regardless of acetate supplementation. High molecular weight triglycerides were more abundant in HG and LG + Ac than in LG and HG + Ac, reflecting a glucose × acetate interaction in which acetate had opposing effects depending on glucose availability.

To identify the individual lipid species driving group separation, random Forest classification was applied to the same four pairwise comparisons on the lipidomic dataset (Figure 5c–f), achieving OOB error rates of 22.2% (HG vs. HG + Ac, Figure 5c), 16.1% (LG vs. LG + Ac, Figure 5d), 5.6% (HG vs. LG, Figure 5e) and 3.8% (HG + Ac vs. LG + Ac, Figure 5f).

In HG versus HG + Ac (Figure 5c), nearly all top discriminators were higher in HG, including 16:0-containing diacylglycerols (DG 37:0, 40:7, 39:0_16:0), triglycerides (TG 36:0_16:0), cholesteryl esters (CE 18:1) and several phosphatidylcholines (PC); only TG 24:2_15:0 and an LPC feature were higher with acetate. LG versus LG + Ac (Figure 5d) was more balanced, with TG 53:19;O3/55:9/54:2_18:1, several PCs, an LPC, phosphatidylethanolamine (PE 38:4) and an L-phosphatidylserine (LPS) feature elevated by acetate, while sphingomyelins (SM 34:1;O3/35:0;O2, 40:1;O2, 34:0;O2) and additional PCs were higher in LG alone.

The glucose contrast without acetate (HG vs. LG, Figure 5e) mirrored panel a, with 16:0-containing DGs and TGs, several PCs and SM 38:0;O2 elevated in HG, and only one feature (PC 40:9/39:2/O-40:2) higher in LG. With acetate present in both conditions (HG + Ac vs. LG + Ac, Figure 5f), the pattern reversed: PE 38:4, TG 53:19;O3/55:9/54:2_18:1, additional PEs, several PCs, CE 18:1 and SM 36:2;O2 were higher in LG + Ac, with only PC 36:6, TG 38:0_16:0 and one PC feature higher in HG + Ac.

Overall, the 16:0-DG/TG compounds drove glucose-level separation without acetate (Figure 5e). However, when HG vs. LG, both in the presence of acetate, the most prominent lipid species separating the treatments were polar lipids, specifically phosphatidylethanolamine (PE, Figure 5f).

## 4. Discussion

The present study aimed to determine how metabolic milieu available for bovine MEC participates in the production of the unique fatty acid composition of dairy fat. For that, glucose and acetate concentrations were modulated in culture to better resemble ruminant plasma, and the metabolic and lipidomic responses were recorded. Results demonstrate that glucose level is a dominant determinant of both the metabolome and lipidome, with acetate modulating the lipidomic and metabolomic profiles depending on the glucose availability. Acetate supplementation to MEC grown on low glucose media, induced the production of medium chain fatty acids and low molecular weight triglycerides, commonly found in bovine milk fat [29]. These changes induced by acetate were accompanied by metabolomics changes consistent with the routing of acetate towards fatty acid synthesis. These findings suggest that the limited capacity of standard MEC cultures to produce milk-like fatty acid profiles reflects the substrate composition of the culture environment rather than an intrinsic limitation of the cells.

The current study model used MAC-T cell line to study the response of bovine MEC to changes in metabolite availability in vitro. More specifically, treatments included varying levels of acetate and glucose, since their levels in commercial media commonly used in bovine MEC studies differ considerably from the in vivo state. Therefore, we hypothesized that changing their concentration will impact the metabolic response of the cells. Nonetheless, other metabolites and nutrients, especially those that change throughout lactation such as long chain fatty acids, propionate and ketone bodies may also contribute to the fatty acid output of the cells.

This model differs from the in vivo setting primarily due to the lack of supporting tissues, such as the mammary gland stroma. The mammary gland stroma includes various cell types which participate in metabolic, and hormonal signaling and hence may affect the ability of the cells to produce dairy fat. For example, fibroblasts that are responsible for the secretion of the extracellular matrix and its modulation during mammogenesis and lactation affect MEC biological functions [30]. Moreover, mammary gland mesenchymal stem cells were shown to change the lipid output of bovine MEC [31]. Nonetheless, to isolate the impact of the metabolites on MEC and to study their ability to respond to the environment, a cell line model provides a controlled system, which is especially important when sensitive methods such as lipidomics and metabolomics are employed. In addition, hormonal treatments in the present study did not include prolactin, in order to reduce the inconsistent role played by the hormone in bovine lactation and lipid synthesis in the mammary gland. Prolactin is usually studied in mice models in the context of milk proteins, with established JAK2-STAT5 activation [32]. However, the role played in lipid biosynthesis pathways in the mammary gland is less established. It has been previously suggested that other endocrine components such as IGF-1 play a role in the activation of lipogenesis upon secretory activation [33]. This is aligned with our results showing that the lipogenic capacity demonstrated in triglyceride accumulation was not altered by the addition of prolactin to the medium. Moreover, the role played by prolactin in bovine lactation is even less established, as prolactin levels in the plasma and lactation gene expression are negatively correlated in at least part of the lactation [34], and prolactin inhibition does not necessarily reduce lactation performance [35]. Consistent with this, lactogenic stimulation that regulates mammary epithelial gene expression and glucose handling in rodent models is not reliably reproduced in bovine MEC, indicating a marked species divergence in prolactin responsiveness [36].

In the current study we used MRM profiling to acquire metabolome and lipidome profiles of the cells. This is an exploratory, semi-targeted screening approach which enables to acquire a large number of compounds without ion fragmentation usually done by LC-MS/MS based methods. To address this limitation, multiple lipid compounds were validated by LC-MS/MS with an authentic internal standard [27]. Structural confirmation was validated by authentic standard co-injection for a targeted subset of metabolites and lipids.

According to the metabolite profile (Figure 1a), both glucose and acetate concentrations gradually separated the samples according to the metabolome, with the two extreme treatments being the high glucose without acetate, which simulated monogastric plasma, and low glucose with acetate, which is closer to ruminant plasma composition. It is possible that the combination of low glucose and high acetate treatment produces a metabolic state that is distinct from the more conventional culture conditions and hence enables better simulation of bovine milk fat synthesis.

Pathway enrichment analysis of differentially abundant metabolites (Figure 2) provided further insight into the metabolic pathways induced by different media conditions. In general, upregulation of metabolic pathways was driven by acetate, whereas down regulation of pathways was driven by glucose (Figure 2a and Figure 2b, respectively). Under high glucose concentration, glucose utilizing pathways were upregulated as reflected in enhanced TCA cycle. Moreover, glycolysis related metabolites (i.e., phosphoenolpyruvate and pyruvate) were also increased, indicating activated glucose utilization. This was not conjugated with lipogenic activity of the cells, reflected by the fact that the lipid biosynthesis pathway was not enhanced. This is in line with previous studies in bovine MEC that have shown glycolysis related genes increase with higher glucose concentrations, without increases in genes related to de novo fatty acid synthesis [37]. On the other hand, low glucose treatments were mostly differentiated from high glucose by higher levels of amino acids (Figure 3b). These may point to a shift to amino acid dominant metabolism when glucose is depleted [38]. The connection between glucose uptake and utilization of amino acids in MEC was demonstrated before [39]. Interestingly, the pathways that dominated the low glucose versus high glucose contrast were no longer prominent when acetate was present in both treatments (LG + Ac versus HG + Ac). In addition, results show that when there are high levels of glucose in the media, addition of acetate was associated with the suppression of TCA cycle (Figure 2b). The addition of acetate to the media may serve as the dominant acetyl-CoA source and reduce the cellular demand for glucose-derived intermediates (Figure 2b). This is consistent with previous findings that adding acetate to MEC decreased the demand for acetyl CoA production through the conversion of glucose to citrate [40]. In addition, the upregulation of pentose phosphate in the high glucose treatment when acetate is present is also correlated to the with the increase in proline (Figure 3d) that is synthesized when NADP is converted to NADPH by the pentose phosphate pathway [41].

Some metabolites were affected differently by acetate supplementation, depending on the glucose background, as demonstrated by a random forest analysis (Figure 3). For example, pantothenic acid, a precursor for CoA, was a top discriminator between high glucose media with or without acetate supplementation (Figure 3a), with a higher abundance in the latter. This could be due to the increased demand for CoA needed to convert the excess acetate provided in the media into acetyl-CoA, leading to a depleted store of pantothenic acid. Another example is citrate concentration which was only decreased by acetate when supplemented to high but not low glucose media (Figure 3a). In addition, caprylic acid (C8:0) was identified as the strongest single discriminator between low glucose media supplemented or not with acetate and was elevated in the acetate-supplemented treatment (Figure 3b). A four-group random forest analysis (Supplementary Figure S3) also revealed that caprylic (C8:0) acid concentration increases progressively across the four treatments, reaching its maximum under low glucose and acetate treatment- the condition most closely approximating ruminant plasma. Caprylic acid is a medium-chain fatty acid characteristic of the bovine milk fat composition that is poorly reproduced in standard MEC culture [42]. The progressive accumulation of caprylic acid as conditions shift toward low glucose and high acetate suggests that MEC have the potential for short and medium-chain fatty acid synthesis in culture, with media conditions limiting this ability.

When adding glucose to low glucose media without acetate present, there was a downregulation of the nicotinate/nicotinamide pathway (Figure 2b) together with suppression of the glycine, serine and threonine metabolism, and cysteine and methionine metabolism. These altered pathways with the addition of glucose may have implications for polar lipid synthesis and modulation of membrane lipids. More specifically, serine provides the headgroup for phosphatidylserine (PS) and ultimately phosphatidylethanolamine (PE) via decarboxylation, and methionine-derived SAM drives the methylation of PE to phosphatidylcholine (PC) [43]. These metabolic shifts may help explain the redistribution of phospholipid species observed in the lipidomic analysis, particularly the higher PE under low glucose with acetate and the higher PC under high glucose conditions.

To complement the metabolomics analysis, we performed a lipidomic analysis of the same four treatment conditions in MEC. The segregation between treatments based on lipidomic profile was dominated by glucose level (Figure 5a). Nonetheless, acetate-supplemented and un-supplemented samples partially overlapped but were shifted relative to one another, indicating that acetate exerts a secondary, glucose-dependent effect on the lipid profile.

The top features discriminating low glucose and low glucose supplemented with acetate were mostly PC species, which were found to be higher when acetate was added to the media (Figure 5d). Random forest classification of high versus low glucose (Figure 5e), identified DG and TG species, containing a 16:0 fatty acid and cholesteryl ester- C18:1 as the strongest discriminators. Most of these features were elevated in high glucose treatment media and depleted in low glucose treatment media, with intermediate behaviour under acetate supplementation and may reflect changes in glucose availability for glycolysis and acetyl-CoA production [44]. Interestingly, when glucose was added to media with acetate, PE species were decreased (Figure 5f). These changes aligned with the metabolomics results demonstrating downregulation of one carbon metabolism and the suppression of methionine and cysteine metabolism in low glucose with acetate treatment.

Total intracellular lipid accumulation increased both by acetate and glucose (Figure 4) aligned with both being precursors for fatty acid synthesis. However, the composition of triglycerides was affected differently. For example, when acetate was added to the media, the abundance of low molecular weight TGs was increased, although this change did not reach statistical significance (Figure 5b). This pattern aligns with the metabolomic finding that caprylic acid, a medium-chain fatty acid of bovine milk fat, accumulated progressively across treatments and reached its maximum under low glucose supplemented with acetate. These results imply that at least for some low molecular weight TG species, accumulation depends on both acetate supplementation and low glucose in the medium, and that the strongest impact on the cells’ ability to produce milk-like lipids requires the combination of both conditions. It should be noted that medium weight TG species (including TG 36:0, TG38:0) were some of the strongest differentiators between treatments with higher concentrations in high glucose media, and under detection levels in low glucose media (Figure 5e). The importance of these lipids and why they are depleted when glucose levels are low, warrants additional investigation. It should be noted that the increased fat accumulation was demonstrated although no change in the lipid biosynthesis pathway was observed. This may suggest a possible interaction between acetate and oleic acid that was used to prime lipid accumulation (Figure 4) and was not added to the samples used for the metabolomic analysis.

Taken together, the metabolomic and lipidomic data suggest that the apparent inability of standard MEC cultures to produce milk-like fatty acid profiles is not necessarily an intrinsic limitation of the cells but a consequence of substrate composition, and that an environment closer to ruminant plasma can partially restore the synthesis and incorporation of short and medium-chain fatty acids into TGs. These findings suggest that substrate composition may be an underappreciated contributor to the limitations of current MEC-based models, and that refining the culture environment may improve their relevance for studying mammary lipid metabolism and advancing cell-based approaches to milk fat production.

## Data Availability

The original contributions presented in this study are included in the article/Supplementary Material. Further inquiries can be directed to the corresponding author.

## Supplementary Materials

The following supporting information can be downloaded at: https://www.mdpi.com/article/10.3390/foods15183234/s1, Figure S1: Acetate dose and time effect on lipogenic gene expression and cell viability; Figure S2: Acetate increases triglyceride accumulation and DGAT expression in MacT cells, with and without prolactin; Figure S3: Four-group Random Forest classification of the MEC metabolome across all treatment conditions.
