# Supplementary material for: Conventional Culture Conditions Limit the Ability of Bovine Mammary Epithelial Cells to Produce Milk Fat
Source: Foods. 2026 Sep 13;15(18):3234. doi: 10.3390/foods15183234 (PMC13606701; doi:10.3390/foods15183234)
Supplement: Supplementary file 1 [file foods-15-03234-s001.zip › foods-4498923-supplementary.pdf]

Supplementary Data File 1

Supplementary Figure S1. Acetate dose and time effect on lipogenic gene expression and cell viability.

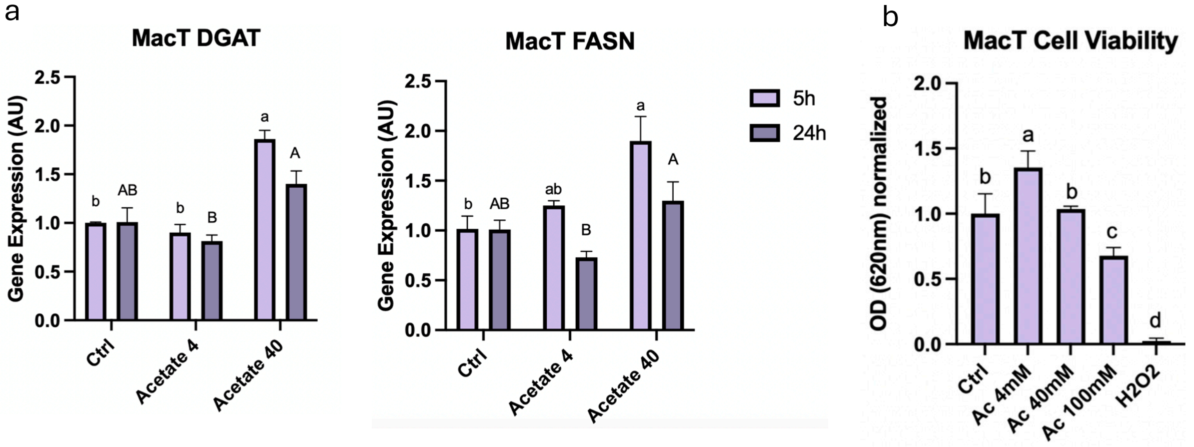

Supplementary Figure S1. Dose and time dependent effects of acetate on lipogenic gene expression and cell viability in MacT cells. Cells were treated with 4 mM, 40 mM, or 100 mM acetate for 5 or 24 hours. (a) mRNA expression of DGAT and FASN after 5- or 24-hour exposure to 4 mM or 40 mM of acetate. (b) Cell viability assessed by XTT assay (Biological industries, Beit Haemek) following 24 hours of 4mM, 40mM or 100 mM of acetate. 5M H_2O_2 was included as a positive control. Bars represent mean ± SEM. Significant differences were determined by one-way ANOVA followed by Tukey-Kramer HSD, different letter indicate significant differences ($P \leq 0.05$).

Supplementary Figure S2. Acetate increases triglyceride accumulation and DGAT expression in MacT cells, with and without prolactin

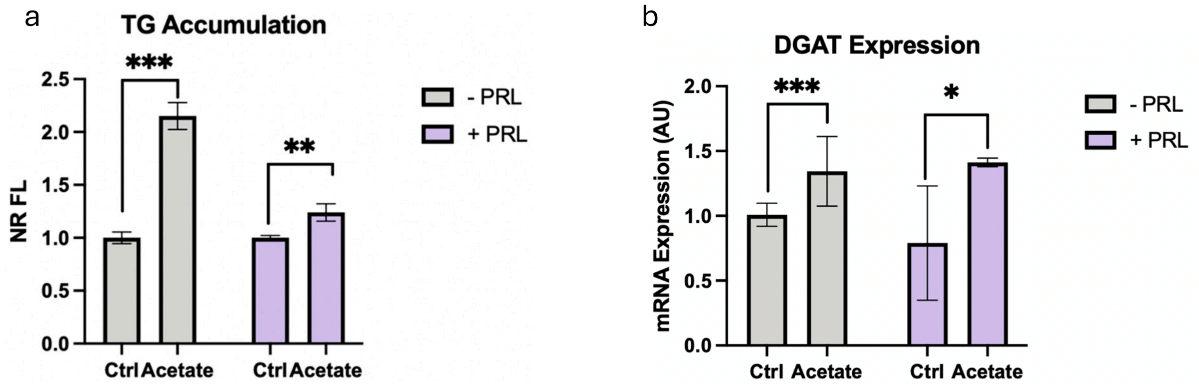

Supplementary Figure S2. Triglyceride accumulation and DGAT mRNA expression in MacT cells treated with acetate (40 mM, 24 h) in media with or without prolactin (1 µg/mL, 48 h). (a) Intracellular triglyceride accumulation, measured as Nile Red fluorescence relative intensity by flow cytometry with control set to 1. (b) DGAT mRNA expression relative with control set to 1. Bars represent mean ± SEM. Significance was determined by Student's t-test (** $P \leq 0.01$, *** $P \leq 0.001$).

Supplementary Figure S3. Four-group Random Forest classification of the MEC metabolome across all treatment conditions

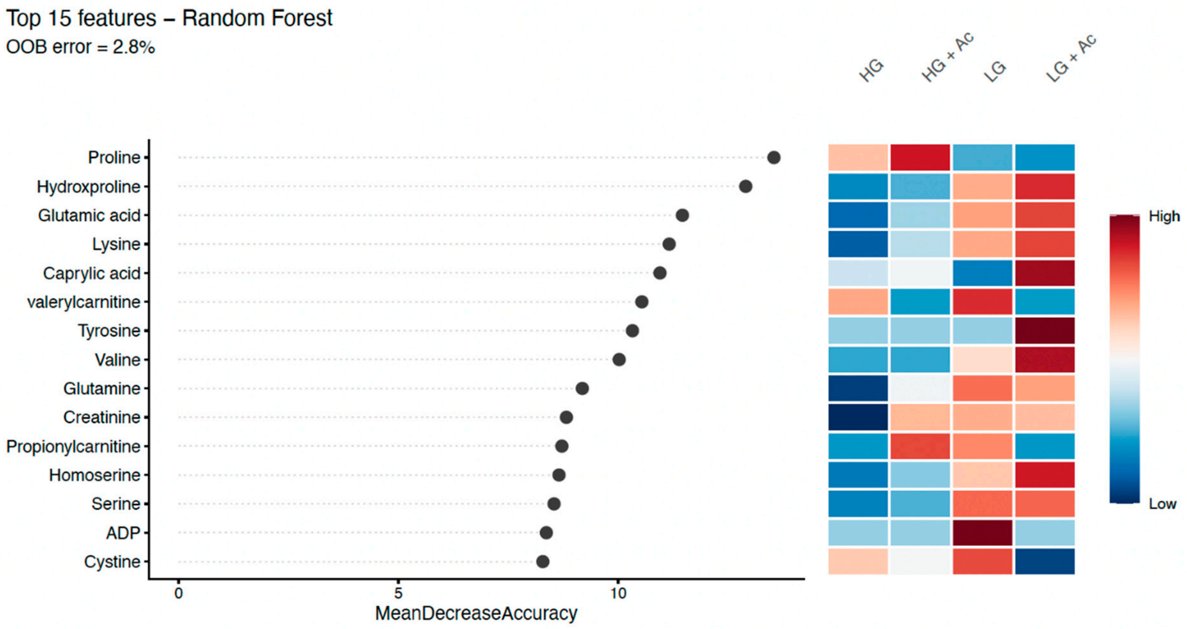

Supplementary Figure S3. Random Forest classification of the metabolomics dataset, showing the top 15 discriminant features ranked by mean decrease in accuracy. The adjacent heatmap displays relative abundance of each feature across the four treatment groups (red = high, blue = low).
